# The Effects of Combined Abiotic and Pathogen Stress in Plants: Insights From Salinity and *Pseudomonas syringae* pv *lachrymans* Interaction in Cucumber

**DOI:** 10.3389/fpls.2018.01691

**Published:** 2018-11-20

**Authors:** Joanna Chojak-Koźniewska, Elżbieta Kuźniak, Janusz Zimny

**Affiliations:** ^1^Genetically Modified Organisms Controlling Laboratory, Plant Breeding and Acclimatization Institute – National Research Institute, Radzików, Poland; ^2^Department of Plant Physiology and Biochemistry, Faculty of Biology and Environmental Protection, University of Lódź, Lódź, Poland; ^3^Department of Plant Biotechnology and Cytogenetics, Plant Breeding and Acclimatization Institute – National Research Institute, Radzików, Poland

**Keywords:** cucumber, *P. syringae*, salt stress, stress interactions, redox signaling, abscisic acid, salicylic acid, carboxylate metabolism

## Abstract

Plants are often challenged by abiotic and biotic stresses acting in combination and the response to combinatorial stress differs from that triggered by each factor individually. Although salinity and pathogens are major stressors limiting plant growth and productivity worldwide, their interaction is poorly understood. The reactions to pathogens overlap with those to abiotic stresses, and reactive oxygen species (ROS) and stress hormones represent central nodes in the interacting signaling pathways. Usually, abiotic stress negatively affects plant susceptibility to disease. Specific focus of this review is on cucumber plants exposed to salt stress and thereafter infected with *Pseudomonas syringae* pv *lachrymans* (*Psl*). We addressed this problem by discussing the changes in photochemistry, the antioxidant system, primary carbon metabolism, salicylic acid (SA) and abscisic acid (ABA) contents. Salt-treated plants were more prone to infection and this effect was determined by changes in the hormonal and redox balance as well as the carboxylate metabolism and activities of some NADPH-generating enzymes. Our detailed understanding of the interactive effects of biotic and abiotic stresses is fundamental to achieve enhanced tolerance to combination stress in agronomically important crops.

## Interactions of Abiotic Stresses and Pathogens: Possible Scenarios

Multifactorial stresses affecting plants are common to many agricultural areas worldwide, and they represent one of the most pressing threats in the field. The well-recognized combinations are those between abiotic stresses (Bandurska and Cieślak, 2013; Rasmussen et al., 2013; Sewelam et al., 2014). With the global climate change, complex stress combinations are expected to occur, and one of the major threats is the establishment of new plant-pathogen interactions due to species migration (Chakraborty, 2005). Our knowledge on how the adverse abiotic conditions can modulate plant-pathogen interactions is limited, except for the plant’s interaction with simultaneous drought and pathogen (Ramegowda and Senthil-Kumar, 2015; Choudhury et al., 2017).

Salinity, affecting many agricultural areas of the globe, and plant pathogens represent an excellent example of abiotic and biotic stresses which co-occur in the field and their interaction may severely influence food quality and safety (Munns and Gilliham, 2015). Salinity (Naliwajski and Skłodowska, 2014; Forieri et al., 2016) and pathogenic bacteria (Gao et al., 2013; Yang et al., 2015) were extensively studied as individual stresses, but their combined impact on crops is not well recognized, although evidence confirming their co-occurrence is still growing (Dileo et al., 2010; Nostar et al., 2013; Nejat and Mantri, 2017; Zhang and Sonnewald, 2017). As to cucumber, the fourth most important vegetable crop worldwide (Lv et al., 2012), 17% of the plants grown in salinated soils in Uzbekistan showed symptoms of *Fusarium solani*-induced diseases (Egamberdieva et al., 2011), and increasing salinity of irrigation water from 0.01 to 5 dS m^-1^ increased the incidence of pythium damping-off of cucumber from 40 to 93% (Al-Sadi et al., 2010).

Reports on combined abiotic and biotic stresses, describe synergistic effects showing that abiotic stress influences the plant-pathogen interaction both positively and negatively, thereby enhancing or decreasing the severity of disease. Most studies with plants simultaneously exposed to drought/heat and biotic stress combinations indicate the dominant role of abiotic factors which facilitates plant diseases (Luo et al., 2005; Kiraly et al., 2008), especially those caused by weakly aggressive facultative pathogens (Desprez-Loustau et al., 2006). Salinity favored disease development caused by *Oidium neolycopersici* in tomato (Kissoudis et al., 2014), increased tomato susceptibility to *Phytophthora infestans* and *Pseudomonas syringae* (Thaler and Bostock, 2004), while it enhanced resistance against *Botrytis cinerea* (Achuo et al., 2006). The comparison of *P. syringae* growth in knockout *Arabidopsis* mutants showed interactions among pathogen growth and physiology and salinity tolerance genes at the gene level (Saleem et al., 2017), but the detailed knowledge of how plant immunity and salt stress tolerance are connected is lacking. Episodic abiotic stress occurring prior to infection was also shown to predispose the plant to disease (Bostock et al., 2014), indicating that responses induced in plants recovering from abiotic stress may conflict with those for resisting pathogens (Boyer, 1995). The increased susceptibility to pathogens under stress may be related to the changed hormonal balance, reduced defense genes expression and to primary metabolism down-regulation which was observed as a general response to multiple stress (Mohr and Cahill, 2003; Prasch and Sonnewald, 2013).

For some interactions, however, the phenomenon of cross-tolerance between abiotic and biotic stresses has been described (Sharma et al., 1996; Achuo et al., 2006; Foyer et al., 2016). It confirms that abiotic and biotic stresses share signals, responsive genes and products and drought improved tomato defense against the *Botrytis cinerea* (Achuo et al., 2006). In a broader context, this phenomenon is linked to defense priming by abiotic factors. The primed plants show relatively little defense expression, but they respond more effectively to the subsequent biotic stress than the non-primed ones due to defense signaling activation (Rejeb et al., 2014). Priming for enhanced defense is interpreted as a defense mechanism with limited fitness costs (Vos et al., 2013). In natural systems, however, the fitness consequences of infection in plants exposed to abiotic stress may vary, as the activation of plant defense can have subsequent effects on the entire plant-associated microbial community, including the non-pathogenic species competing the disease-inducing ones (Barrett et al., 2009; Vos et al., 2013).

## Convergence Points and Master Regulators of the Interaction Between Abiotic Factors and Pathogens

Stress signaling in plants constitutes a complex network (Miller et al., 2009; Baxter et al., 2014; Gilroy et al., 2014), however, the main crosstalk nodes between abiotic and biotic signaling pathways have been identified. They are represented by signaling components shared between abiotic and biotic stress responses, such as ROS and redox compounds, calcium ions, phytohormones as well as signal-response coupling factors, e.g., protein kinases and transcription factors (Fujita et al., 2006).

At the sites of stress perception, the information of the nature of the stimulus is encoded by the spatiotemporal dynamics in ROS and calcium [Ca^2+^] cellular changes (Fujita et al., 2006). These ROS and calcium signatures can be decoded by different sensors, leading to stimulus-specific hormonal and metabolic response at the site of stress action (Fujita et al., 2006; Tippmann et al., 2006). This stress-specific response is usually transmitted throughout the entire organism to elicit an integrated whole plant reaction. Stresses, including salinity and pathogen infection, can be signaled by cell-to-cell autopropagating, vascular ROS and calcium waves which are integrated by the respiratory burst oxidase homolog (Rboh) protein, and by the activation of stress-specific metabolic cues (Jiang et al., 2012; Gilroy et al., 2014).

The ROS signature is shaped by the antioxidant system, with the ascorbate-glutathione (AA-GSH) cycle being the major ROS-processing mechanism which links the protection against ROS to redox-regulated defense (Kuźniak, 2010; Foyer and Noctor, 2011; Shigeoka and Maruta, 2014). However, there are only some reports on the involvement of AA-GSH cycle components in plant tolerance to concurrent abiotic and biotic stresses refer mainly to example ascorbate peroxidase (APX, Satapathy et al., 2012; Nenova and Bogoeva, 2014) or APX and glutathione reductase activities under salt stress and fungal infection (Nostar et al., 2013).

Similarly to ROS, phytohormones are important players in orchestrating signaling pathways as well as transcriptional and metabolic responses shared between abiotic and biotic stresses (Robert-Seilaniantz et al., 2011). SA, jasmonic acid (JA) and ethylene (ET) are mainly known to control plant defense against pathogens (Pieterse et al., 2009; Verma et al., 2016). ABA primarily regulates plant responses to drought, low temperature and salinity (Sah et al., 2016), but it also mediates defense against pathogens. Treatment with ABA increases plant susceptibility to bacterial and fungal pathogens, and inhibition of ABA signaling improves plant defense against pathogens (Audenaert et al., 2002; Mohr and Cahill, 2003; Anderson et al., 2004; Asselbergh et al., 2007). ABA usually antagonizes SA and JA/ET defense signaling thereby interfering with plant responses to biotrophic and necrotrophic pathogens, respectively (Ton et al., 2009; Lievens et al., 2017). At the pre-invasion stage, however, ABA can have a positive effect on defense, as it increases penetration resistance by inducing stomatal closure (Ton et al., 2009).

Under combined stress, ABA is recognized as the master hormonal switch prioritizing the abiotic or biotic responses, depending on the nature of individual stresses and the harmful effects they can induce in plants. Two ABA signaling components, the transcription factor ATAF1 (*Arabidopsis* NAC domain containing 1) and the proline oxidase ERD15 (EARLY RESPONSIVE TO DEHYDRATION 15) act as switches which activate ABA-dependent, post-penetration resistant responses at the expense of abiotic stress tolerance (Ton et al., 2009; Atkinson and Urwin, 2012; Suzuki et al., 2014).

The close relationship of ABA with SA and JA/ET-mediated defense signaling may have critical consequences for the outcome of plant-pathogen interaction in the field, as any abiotic stress which leads to ABA accumulation is likely to suppress disease resistance mechanisms (Sivakumaran et al., 2016). The outcome of plant-pathogen interaction can be also modulated by crosstalk between ROS and ABA signaling. Salinity predisposing plants to disease has been found to increase ROS content by impairing the ROS-scavenging system (Jiang and Deyholos, 2006), and ABA-regulated genes are also induced by oxidative stress (Cho et al., 2009).

In the multistress environment, the interactions between the signaling pathways provide the plant with powerful regulatory capacity and are likely to reduce the metabolic costs of plant defense (Vos et al., 2013). Crosstalk between abiotic and biotic stresses leads to changes in the primary metabolism which is shifted from growth and biomass production programs to defensive processes (Rojas et al., 2014). By understanding these cross-regulation mechanisms, we could predict the outcome of plant-pathogen interactions under abiotic stress conditions. It is especially important in the context of global climate changes, when the abiotic and biotic stresses are expected to increase.

## Case Study: Cucumber Response to Sequential Salt Stress and Bacterial Pathogen Infection

As the interaction between salinity and pathogens is still poorly recognized, we discuss here how salt stress influences the plant response to diseases, taking leads from studies on cucumber sequentially exposed to salt stress and angular leaf spot (ALS), the second most severe cucumber disease, caused by *Psl* (Olczak-Woltman et al., 2009).

In cucumber exposed to 7-day salt stress and thereafter inoculated with *Psl* the combinatorial stress intensified the negative impact of NaCl on plant growth, confirming results on the additive effect of stress factors. Salt-treated cucumber was more prone to ALS as shown by enhanced bacteria growth and disease symptom development in the NaCl-treated plants simultaneously with the recovery from salt stress (Chojak-Koźniewska et al., 2017). Salt stress and recovery are mediated by shared signaling components, including ROS and redox elements, phytohormones, and C metabolites which might also contribute to pathogen defense (Tang et al., 2015).

**FIGURE 1:**
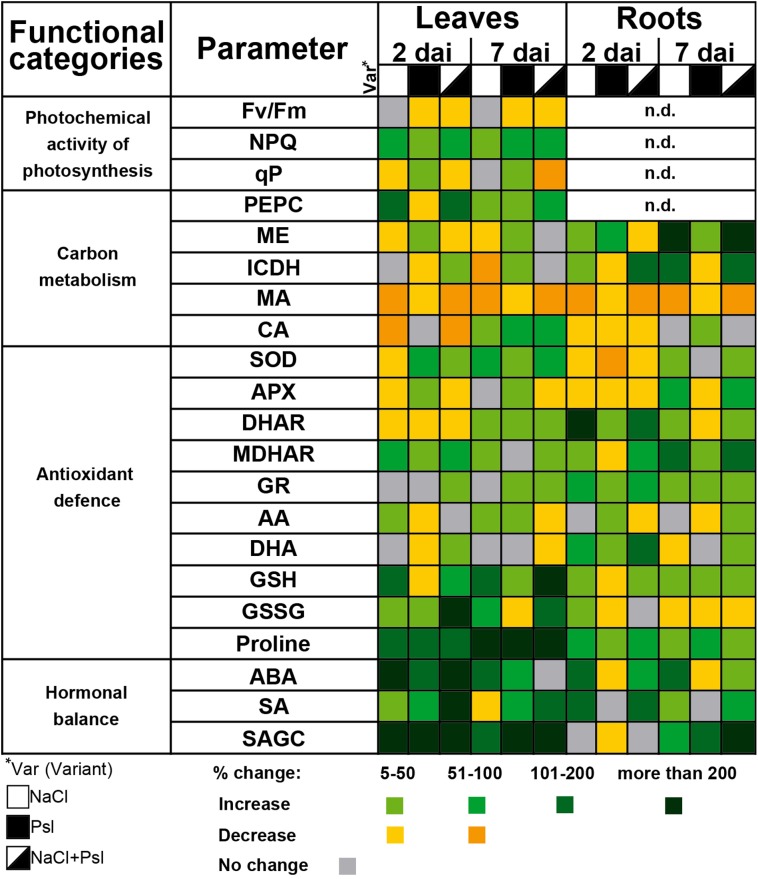
The effect of salt stress and *Pseudomonas syringae* pv *lachrymans* (Psl) infection applied individually and in combination on photochemical activity of photosynthesis, carbon metabolism, antioxidant defense and hormonal balance in leaves and roots of cucumber plants (Chojak et al., 2012; Chojak-Koźniewska, 2017; Chojak-Koźniewska et al., 2017, 2018). Plants were pretreated for 7 days with 100 mM NaCl and then infected with *Psl*. Analyses were performed 2 and 7 days after inoculation (dai). Changes in contents/activities are *color coded*, relative to control set as 100%. Fv/Fm, maximum PSII quantum yield; NPQ, non-photochemical quenching; qP, photochemical quenching coefficient; PEPC, phosphoenolpyruvate carboxylase; ME, NADP-malic enzyme; ICDH, NADP-isocitrate dehydrogenase; MA, malic acid; CA, citric acid; SOD, superoxide dismutase; APX, ascorbate peroxidase; DHAR, dehydroascorbate reductase; MDHAR, monodehydroascorbate reductase; GR, glutathione reductase; AA, ascorbic acid (reduced); DHA, dehydroascorbate; GSH, glutathione (reduced); GSSG, glutathione disulphide; ABA, abscisic acid; SA, free salicylic acid; SAGC, SA conjugates with glucose.

In plants, stress sensing is primarily reflected in PSII photochemistry imbalance, and maximum PSII quantum yield (Fv/Fm) and NPQ are recognized indicators of plant stress (Kuźniak et al., 2010; Qu et al., 2012). Changes in the photosynthetic apparatus also play an important regulatory role via retrograde signaling (Gollan et al., 2015). In cucumber sequentially exposed to NaCl and *Psl*, Fv/Fm and photochemical quenching coefficient (qP) which quantify the photochemical efficiency, decreased, and the single stress scenarios of perturbations of PSII were changed under combined stress, e.g., qP was significantly decreased only in plants exposed to NaCl and Psl (Chojak-Koźniewska et al., 2018). The interaction of NaCl and *Psl* strongly reduced the cucumber capacity to recover leaf photochemistry after salt stress alleviation when compared to plants subjected to single stress (Figure 1). This could be caused by impaired photosynthetic biochemistry (Ennahli and Earl, 2005) or decreased CO_2_ availability due to sustained stomata closure (Galmés et al., 2007). The salt stress-induced stomata closure signaled by root-produced ABA was intensified after infection, likely via SA/H_2_O_2_-related mechanism, and could contribute to the prolonged inhibition of PSII under combined stress (Chojak-Koźniewska et al., 2017).

In cucumber, salt stress, *Psl* and their combination induced NPQ which protects plants from damaging effects of ROS and integrates into the defense responses against biotic stress (Chojak-Koźniewska et al., 2018). In *Arabidopsis*, increased photooxidative stress at PSII decreased resistance to *Sclerotinia sclerotiorum* (Zhou et al., 2015) and plants impaired in NPQ were suggested to be constantly primed to pathogen attack (Barczak-Brzyżek et al., 2017). Many authors reported infection-induced inhibition of photosynthesis, but its role in pathogenesis remains unclear as it could reflect the attack strategy of the pathogen and a defense response relying on reprogramming plant metabolism from growth to defense (Cheng et al., 2016; Dong et al., 2016).

In cucumber coping with combinatorial stress, the infection-induced oxidative stress, manifested by H_2_O_2_ accumulation and lipid peroxidation, was stronger than in plants exposed to salinity and *Psl* individually (Chojak et al., 2012). The non-halo lesion type similar to the hypersensitive response is an important component of cucumber resistance to ALS and chlorotic halo is typical for susceptibility (Słomnicka et al., 2018). Thus, ROS generated in the infected plants contributed to susceptibility to ALS. The antioxidant response was organ and stimulus-specific, except for proline accumulation (Figure 1) which is a common defense response in plants grown under stress (Nostar et al., 2013; Pandey et al., 2015). Other studies also showed proline accumulation after salt treatment and bacterial pathogen infection, correlated with disease severity (El-Hendawy, 1999; Tiwari et al., 2010). In roots, the activities of superoxide dismutase (SOD) and the AA-GSH cycle in plants sequentially exposed to salt stress and *Psl*, closely resembled those induced by salinity, confirming abiotic stress dominant impact of. In leaves, the combinatorial stress-induced changes showed that plants previously exposed to salt stress required specific antioxidant protection after infection, as exemplified by the induction of chloroplastic FeSOD found exclusively in plants under combined stress (Chojak-Koźniewska et al., 2017). 7 days after infection, during the salt stress recovery phase (Figure 1), the AA-GSH cycle-related antioxidant profile in the leaves of plants exposed to NaCl and *Psl* was characterized by significantly decreased APX activity and ascorbic acid (AA) content, accompanied by glutathione (GSH) accumulation (Chojak-Koźniewska, 2017). This indicated a substantial uncoupling of ascorbate and glutathione redox pairs under combined stress. Contrary, NaCl treatment and bacterial infection applied individually induced parallel changes in ascorbate and glutathione pools. In other studies, NaCl led to a decrease in ascorbate and glutathione levels in cucumber seedlings and *P. syringae* infection increased the contents of these antioxidants in *Arabidopsis* (Großkinsky et al., 2012; Shu et al., 2013). As redox signals are early warnings controlling the adjustment of energy production to consumption in the leaf (Foyer and Noctor, 2009), these changes could regulate the execution of plant metabolic reprogramming under combined stress. The ascorbate pool and the related components of the AA-GSH cycle were affected stronger than glutathione (Chojak-Koźniewska, 2017). Ascorbate plays a critical regulatory role in the network of photosynthesis, respiratory electron transport and tricarboxylic acid cycle (Szarka et al., 2013), thus these changes could have negative consequences for plant resistance to *Psl*.

Besides the redox modifications, salt stress through ABA upregulation had antagonistic effects on SA-mediated signaling and compromised the defense against the pathogen. The changed ABA/SA equilibrium in leaves hindered *PR1* gene expression (pathogenesis-related 1), known to regulate SA-mediated defense to pathogens (Rivas-San Vicente and Plasencia, 2011; Chojak-Koźniewska et al., 2017; Zhang and Sonnewald, 2017).

Stress defense responses and recovery require energy inputs and diversion of carbon metabolites and reducing equivalents to anabolic pathways. NADPH-generating enzymes, such as NADP-isocitrate dehydrogenase (ICDH) and NADP-malic enzyme (ME), provide NADPH which is required for growth and detoxification, participates in the equilibrium of cellar redox homeostasis, supports the AA-GSH cycle and the NADPH oxidase in the apoplast (Noctor, 2006; Leterrier et al., 2012). Abiotic stress, which affected plants stronger than infection, was the dominant factor shaping the response of carbon metabolism to combinatorial stress at both the biochemical and transcriptomic levels (Chojak-Koźniewska et al., 2018). In roots, salt stress intensified the activities of ME and ICDH, indicating the need for increased detoxification. Although in other studies similar results were also reported for leaves of salt-stressed cucumber (Hýsková et al., 2017), we observed that the NADPH-generating enzyme activities tended to decrease (Figure 1). This, combined with the reduced contents of malic and citric acids which are involved in energy-producing pathways (López-Millán et al., 2000) and compensate for salt-stress induced ionic imbalance (Sanchez et al., 2008), could represent changes in the leaf metabolic environment predisposing plants to infection. Decreased contents of malic and citric acids in salt-stressed cucumber were also described by Zhong et al. (2016). As to the biotic stress, high level of malic acid was suggested to be a pre-selection criterion for resistance to ascochyta blight in chickpea (Çağirgan et al., 2011).

The integration of stress response at the whole-plant level requires long distance signals, including ROS, hormones and metabolites which communicate the information of the status of roots to leaves and vice versa (Mittler et al., 2011; Gilroy et al., 2014; Ko and Helariutta, 2017). Biotic stress in leaves initiated rootward signaling to induce whole-plant stress response as shown by specific changes in the ABA/SA balance, carbon metabolism and the profiles of antioxidants in roots of plants which leaves were infected with *Psl*. Similarly, abiotic stress sensed in roots prior to infection changed the status of leaves during recovery, making them more prone to infection (Figure 1).

**FIGURE 2:**
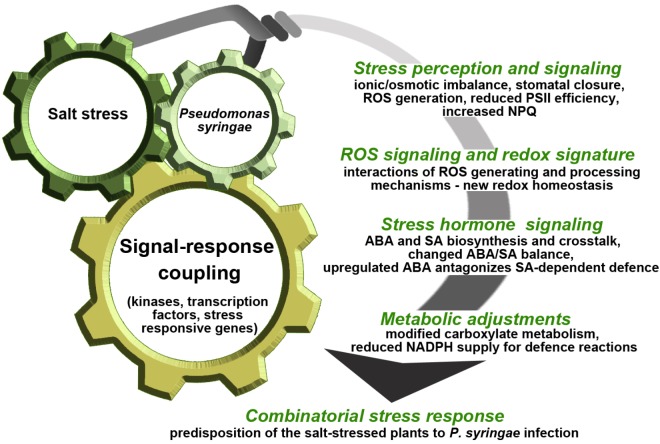
Schematic illustration of cucumber plant response to sequential application of salt stress and *P. syringae*. Salt stress and *P. syringae* infection differ in severity of impact on cucumber plants and the combinatorial stress response is dominated by the abiotic factor. Episodic salt stress occurring prior to infection may predispose cucumber plants to *P. syringae* by weakening SA-mediated defense as well as by shaping the hormonal, ROS/redox and metabolic signals induced under combinatorial stress. These signals are transduced by specific kinases and transcription factors activating or suppressing functional genes, finally resulting in higher susceptibility of the salt-stressed plants to *P. syringae* infection. This model is based on data presented in Chojak-Koźniewska (2017) and Chojak-Koźniewska et al. (2017, 2018). PSII, photosystem II; NPQ, non-photochemical quenching; ROS, reactive oxygen species; ABA, abscisic acid; SA, salicylic acid; NADPH, nicotinamide adenine dinucleotide phosphate.

In cucumber, salinity and bacterial pathogen applied sequentially generated prolonged inhibition of PSII, and unique redox signature as a result of ROS overproduction and novel interactions between the AA-GSH cycle components. This implies specific adjustments to other signaling components, especially ABA and SA. Salt stress-induced ABA accumulation compromised the SA-mediated defense response. This was favored by modifications in the carboxylate metabolism and could lead to insufficient energy and reducing power supply to defense reactions (Chojak-Koźniewska, 2017; Chojak-Koźniewska et al., 2017, 2018). This example supports the role of ABA in predisposition, and illustrates biochemical mechanisms underpinning this phenomenon in the context of whole-plant response (Figure 2). The case study, supported by additional data, demonstrated that under combined stress most single stress responses were maintained, although differentially regulated. The functional analysis of the enhanced or reduced components of the stress responses may be a hint to what processes and in what stress scenarios could increase plant tolerance.

## Concluding Remarks

Stress factors acting in combination lead to decreases in crop yield that exceed single stresses. Plant acclimations to combinatorial stress vary widely depending on the type, sequence of application, and intensity of the individual stresses implied. Many studies have shown that combined stress triggers specific transcriptomic response. Yet, little is known about the stress combination-unique response elicited by the partially overlapping stressors at the physiological and metabolic levels. The response to a combination of abiotic and biotic stresses is usually dominated by abiotic stress, at the expense of resistance to pathogens. However, our current understanding of mechanisms predisposing plants affected by abiotic stress to infectious diseases is still limited.

The knowledge of how abiotic environmental factors influence plant resistance to pathogens and of processes specifically involved in response to combined abiotic and biotic stress has implications for disease management and is important with respect to the breeding programs aimed at improving multiple stress tolerance in plants.

## Author Contributions

JC-K, EK, and JZ took responsibility for the integrity of the work as a whole.

## Conflict of Interest Statement

The authors declare that the research was conducted in the absence of any commercial or financial relationships that could be construed as a potential conflict of interest.
